# Case of Recovery from the Passage of an Iron Bar through the Head

**Published:** 1850-08

**Authors:** 


*Case of Recovery from the passage of an iron bar through the head*—

Our readers will recollect the account of the passage of an iron bar through the head, and the recovery of the patient, which was copied into this Journal several months ago. In the *American Journal of the Medical Sciences*, for July, 1850, we find a communication on the case from the pen of Henry J. Bigelow, M. D., Prof. of Surgery in Harvard University, which we copy, excluding some letters and affidavits relative to the circumstances attending and following the accident, together with the notes of the daily symptoms of the case.—EDITOR BUFFALO MEDICAL JOURNAL.

The following case, perhaps unparalleled in the annals of surgery, and of which some interesting details have already been published, occurred in the practice of Dr. J. M. Harlow, of Cavendish, Vermont. Having received a verbal account of the accident, a few days after its occurrence, from a medical gentleman who had examined the patient, I thus became incidentally interested in it ; and having since had an opportunity, through the politeness of Dr. Harlow, of observing the patient, who remained in Boston a number of weeks under my charge, I have been able to satisfy myself of the occurrence and extent of the injury, as of the manner of its infliction. I am also indebted to the same gentleman for procuring at my request the testimony of a number of persons who were cognizant of the accident or its sequel.

Those who are skeptical in admitting the co-existence of a lesion so grave, with an inconsiderable degree of disturbance of function, will be interested in further details connected with the case; while it is due to science that a more complete record should be made of so remarkable an injury.

The accident occurred on the line of the Rutland and Burlington Railroad, on the 13th of September, 1848. The subject of it, Phineas P. Gage, is of middle stature, twenty-five years of age, shrewd and intelligent. According to this statement, he was charging with powder a hole drilled in a rock, for the purpose of blasting. It appears that it is customary in filling the hole to cover the powder with sand. In this case, the charge having been adjusted, Mr. Gage directed his assistant to pour in the sand; and at the interval of a few seconds, his head being averted, and supposing the sand to have been properly placed, he droped the head of the iron as usual upon the charge to consolidate or "*tamp it in.*" The assistant had failed to obey the order, and the iron striking fire upon the rock, the uncovered powder was ignited and the explosion took place. Mr. Gage was at this time standing above the hole, leaning forward, with his face slightly averted; and the bar of iron was projected directly upwards in a line of its axis, passing completely through his head and high into the air. The wound thus received, and which is more fully described in the sequel, was oblique, traversing the cranium in a straight line from the angle of the lower jaw on one side to the centre of the frontal bone above, near the sagittal suture, where the missile emerged; and the iron thus forcibly thrown into the air was picked up at a distance of some rods from the patient, smeared with brains and blood.

From this extraordinary lesion, the patient has quite recovered in his faculties of body and mind, with the loss only of the sight of the injured eye.

The iron which thus traversed the skull weighs thirteen and a quarter pounds. It is three feet seven inches in length, and one and a quarter inches in diameter. The end which entered first is pointed; the taper being seven inches long, and the diameter of the point one quarter of an inch; circumstances to which the patient perhaps owes his life. The iron is unlike any other, and was made by a neighboring blacksmith to please the fancy of the owner.

Dr. Harlow, in the graphic account above alluded to, states that "immediately after the explosion the patient was thrown on his back, and gave a few convulsive motions of the extremities, but spoke in a few moments. His men (with whom he was a great favorite) took him in their arms and carried him to the road, only a few rods distant, and sat him into an ox cart, in which he road, sitting erect, full three quarters of a mile, to the hotel of Mr. Joseph Adams, in this village. He got out of the cart himself, and with a little assistance walked up a long flight of stairs, into the hall, where he was dressed."

The leading feature of the case is its improbability. A physician who holds in his hand a crowbar, three feet and a half long, and more than thirteen pounds in weight, will not believe that it has been driven with a crash through the brain of a man who is still able to walk off, talking with composure and equanimity of the hole in his head. This is the sort of accident that happens in the pantomime at the theatre, but not elsewhere. Yet there is every reason for supposing it in this case literally true. Being at first wholly skeptical, I have been personally convinced; and this has been the experience of many medical gentlemen who, having first heard of the circumstances, have had subsequent opportunity to examine the evidence.

This evidence is comprised in the testimony of individuals, and in the anatomical and physiological character of the lesion itself.

The above accounts from different individuals, concur in assigning to the accident a common cause. They are selected as the most complete among about a dozen of similar documents forwarded to me by Dr. Harlow, who was kind enough to procure them at my request; and which bear the signature of many respectable persons in and about the town of Cavendish, and all corroborative of the circumstances as here detailed. The accident occurred in open day, in a quarry in which a considerable number of men were at work, many of whom were witnesses of it, and all of whom were attracted by it. Suffice it to say, that in a thickly populated country neighborhood, to which the facts were matter of daily discussion at the time of their occurrence, there is no difference of belief, nor has there been any doubt that the iron was actually driven through the brain. A considerable number of medical gentlemen also visited the case at various times to satisfy their incredulity.

Assuming the point that the wound was the result of a missile projected from below upwards, it may be asked whether the wound might not have been made by a stone, while the bar was at the same moment thrown into the air. It may be replied in answer, that the rock was not split, nor, as far as could be learned, disintegrated. Besides, an angular bit of stone, would have been likely to have produced quite as much laceration as the bar of iron; and it is in fact possible that the tapering point of the latter divided and repelled the soft parts, especially the brain, in a way that enabled the smooth surface of the iron to glide through with less injury. And assuming the only possible hypothesis, that the round bar followed exactly the direction of its axis, the missle may be considered as a sphere of one and a quarter inches diameter, preceded by a conical and polished wedge.

The patient visited Boston in January, 1850, and remained some time under my observation, during which he was presented to a meeting of the Boston Society for Medical improvement, and also to the medical class at the hospital. His head, now perfectly healed, exhibits the following appearances.

A linear cicatrix of a inch in length occupies the left ramus of the jaw near its angle. A little thickening of the soft tissues is discovered about the corresponding malar bone. The eyelid of this side is shut, and the patient unable to open it. The eye considerably more prominent than the other, offers a singular confirmation of the points illustrated by the prepared skull described below. It will there be sesn that the parts of the orbit necessarily cut away are those occupied by the levator palpebræ superioris, the levator oculi, and the the abducens muscles. In addition to a ptosis of the lid, the eye is found incapable of executing either the outward or upward motion ; while the other muscles animated by the motor communis are unimpared. Upon the head, and covered by hair, is a large unequal depression and elevation. A portrait of the shaved head is given in the plate; and it will be seen that a piece of cranium about the size of the palm of the hand, the posterior border lying near the coronal suture, its anterior edge low upon the forehead, was raised upon the latter as a hinge to allow the egress of the bar; and that it still remains raised and prominent. Behind it is an irregular and deep sulcus several inches in length, beneath which the pulsations of the brain can be perceived.

In order to ascertain how far it might be possible for this bar of an inch and a quarter diameter to to traverse the skull in the track assigned to it, I procured a common skull, in which the zygomatic arches are barely visible from above; and having entered a drill near the left angle of the lower jaw, passed obliquely upwards to the median line of the cranium just in front of the junction of the sagittal and coronal sutures This aperture was then enlarged until it allowed the passage of the bar in question, and the loss of substance strikingly corresponds with the lesion said to have been received by the patient. From the coronoid process of the lower jaw is removed a fragment measuring about three-fourths of a inch in length. This fragment in the patient's case might have been fractured and subsequently reunited.

The hole now enters obliquely beneath the zygomatic arch, encroaching equally upon all its walls. In fact, it entirely occupies this cavity; the posterior wall of the antrum being partially excavated at the front of the hole, the whole orbitar portion of the sphenoid bone being removed behind, as also the anterior part of the squamouse portion of the bone, and the internal surface of the zygoma and malar bone laterally. In the orbit, the sphenoid bone, part of the superior maxillary below, and a large portion of the frontal above, are cut away, and with these fragments much of the spheno-maxillary fissure; leaving, however, the optic foramen intact about a quarter of an inch to the inside of the track of the bar.

The base of the skull upon the inside of the cranium presents a cylindrical hole of an inch and a quarter diameter, and such as may be described by a pair of compasses, one leg of which is placed upon the lesser wing of the sphenoid bone at an eighth of an inch from its extremity, cutting the frontal, temporal and sphenoid bones; the other, half a inch outside the internal optic foramen.

The calvaria is traversed by a hole, two-thirds of which is upon the left, and one-third upon the right of the median line, its posterior body being quite near the coronal suture. The iron freely traverses the oblique hole thus described.

It is obvious that a considerable portion of the brain must have been carried away; that while a portion of its lateral substance may have remained intact, the whole central part of the anterior lobe, and the front of the sphenoidal or middle lobe must have been lacerated and destroyed.—This loss of substance would also lay open the anterior extremity of the left lateral ventricle; and the iron, in emerging from above must have impinged upon the right cerebral lobe, lacerating the falx and the longitudinal sinus. Yet the optic nerve remained unbroken in the narrow interval between the inner wall of the orbit. The eye, forcibly thrust forward at the moment of the passage, might again have receded into its socket, from which it was again somewhat protruded during the subsequent inflammation.

It is fair to suppose that the polished conical extremity of the iron which first entered the cavity of the cranium prepared the passage for the thick cylindrical bar which followed; and that the point, in reaching and largely breaking open the vault of the cranium, afforded an ample egress for the cerebral substance, thus preventing compression of the remainder.

Yet it is difficult to admit that the aperture could have been thus violently forced through without a certain comminution of the base of the cranium driven inwards upon the cerebral cavity.

Little need be said of the physiological possibility of this history. It is well known that considerable portions of the brain has been in some cases abstracted without impairing its functions. Atrophy of an entire cerebral hemisphere has also been recorded.

But the remarkable features of the present case lie not only in the loss of cerebral substance, but also the singular chance which exempted the brain from either concussion or compression; which guided the enormous missile exactly in the direction of its axis, and which averted the dangers of subsequent inflammation. An entire lung is often disabled by disease; but I believe that there is no parallel to the case in the Hunterian collection of a lung and thorax violently transfixed by the shaft of a carriage.

Taking all the circumstances into consideration, it may be doubted whether the present is not the most remarkable history of injury to the brain which has been recorded.*

---

* The iron bar has been deposited in the museum of the Massachusetts Medical College, where it may be seen, together with a cast of the patient's head.

